# *In silico* prediction of the impact of genomic variations in the small conductance calcium activated potassium channel SK3 structure and function

**DOI:** 10.3389/fnins.2025.1631536

**Published:** 2025-10-10

**Authors:** Lucia Padilla, Coral Del Val, Daria B. Neidre, Agustín S. Kokenge, Juan E. Martinez, Antonio L. Teixeira, Igor Zwir, Gabriel A. de Erausquin

**Affiliations:** 1 Laboratory of Brain Modulation and Repair, Glenn Biggs Institute for Alzheimer’s’ and Neurodegenerative Diseases, University of Texas Health San Antonio, San Antonio, TX, United States; 2 Department of Computer Science and Artificial Intelligence, Andalusian Research Institute in Data Science and Computational Intelligence (DaSCI), University of Granada, Granada, Spain; 3 Instituto de Investigación Sanitaria ibs. GRANADA, Hospitales Universitarios de Granada-Universidad de Granada, Granada, Spain; 4 Department of Psychiatry, Washington University, St. Louis, MO, United States

**Keywords:** SK3 channels, neurons, dopaminergic neurons, brain development, neurodegeneration

## Abstract

The small-conductance calcium-activated potassium channel SK3, encoded by the KCNN3 gene, plays a critical role in regulating dopaminergic neuron (DN) firing patterns by modulating after hyperpolarization currents. SK3 dysfunction has been implicated in neuropsychiatric and neurodegenerative disorders. We analyzed structural and functional consequences of KCNN3 splicing and genetic variation. Alternative splicing variants of the KCNN3 gene were retrieved from the Ensembl database and aligned using T-Coffee, manually inspected and curated. Protein domains were identified with Pfam 35.0, SMART 9.0, and InterPro 98.0, and visualized. An AlphaFold2 model of SK3 full-length protein (UniProt: Q9UGI6) used as reference and structural models of its splicing variants were predicted with ColabFold. Functional domains (S1–S6 transmembrane helices, H5 pore loop, and calmodulin-binding) were defined and superimposed onto the AlphaFold2 reference. Domain integrity was assessed based on completeness of all expected residue indices within each functional region. SNPs and CNVs across all coding KCNN3 splicing variants were analyzed, classified, and filtered to isolate pathogenic variants prioritizing non-synonymous amino acid substitutions. Differential variant impacts across splicing isoforms were assessed by mapping variant positions to individual transcript protein sequences and used to predict functional consequences. Two long and two short splicing variants are known. Short variants lack the motif required for potassium channels. Pathogenic variants result from missense mutations resulting in amino acid substitutions. In all cases, the consequential effects depend on the specific location and role of the amino acid being changed.

## Introduction

During embryonic development in humans, dopaminergic neurons DN are formed in the mesencephalon and differentiate into three major clusters corresponding to the Retrobural Field (RRF), Substantia Nigra pars compacta (SNc) and Ventral Tegmental Area (VTA) respectively (Hegarty et al., 2013). DN *in vivo* typically fire either regularly, in a pacemaker fashion, or in bursts (Grace and Bunney, 1984a, 1984b; Shepard & German, 1988a). Pacemaker activity produces a tonic release of dopamine (Grace, 1991; Overton and Clark, 1997), while burst firing results in phasic release of dopamine in terminal axonal fields (Bean and Roth, 1991; Gonon and Buda, 1985). When partially denervated in brain slices, DN only maintain pacemaker activity indicating that burst firing is driven by synaptic input (Kita et al., 1986; Sanghera et al., 1984). Bursts consists of 3–7 action potentials that progressively decrease in amplitude and increase in duration, followed by a pause. Non-burst firing patterns, whether pacemaking or not, consist of similarly shaped spikes (Grace and Bunney, 1984a, 1984b; Gu et al., 1991; Shepard & German, 1988b). The controlling switch between regular and burst firing patterns is the small conductance potassium channel sensitive to calcium (SK) (Gu et al., 1991) which is the subject of this work.

There are four subfamilies of SK channels: SK1, SK2, SK3 and the intermediate conductance channel, IKCa1. The latter is encoded by the gene KCNN4 which is not expressed in neurons and therefore will not be discussed further (González et al., 2012). On the other hand. SK1 and SK2 channels are highly expressed in the hippocampus and cortex, whereas SK3 channels are abundant in the thalamus and hypothalamus and have the highest expression levels in the midbrain (Sah and Faber, 2002). All SK channels are voltage independent but open in response to nanomolar changes in cytosolic free calcium (Park, 1994); the ensuing outward potassium current contributes to afterhyperpolarization after a single spike event as well as in the pacemaker properties of neurons (Herrik et al., 2010; Ji et al., 2009; Ji and Shepard, 2006; Wolfart and Roeper, 2002). Following an action potential, potassium currents cause an after-hyperpolarization event (AHP) consisting of fast, medium and slow components (Lancaster and Nicoll, 1987; Sah, 1996; Storm, 1987, 1990). In midbrain DNs, SK3 channels mediate the medium AHP component and control firing frequency (Seutin and Liégeois, 2007; Waroux et al., 2005; Wolfart and Roeper, 2002). Action potential-caused depolarization causes influx of ionized calcium into the cytosol via voltaje dependent calcium channels; further release from intracellular calcium stores is mediated by calcium-induced calcium release receptors (ryanodine receptors) leading to the activation of SK3 channels (Blatz and Magleby, 1986; Park, 1994) and Kd below 1 μmol/L (Halling et al., 2022). SK3 channels do not have a calcium-binding motif; instead, gating is mediated by calmodulin binding to the heteromeric complex of four pore-forming *α* subunits (Lee et al., 2003). Adult SNpc neurons richly express SK3 mRNAs, whereas DN in the VTA do so much less, and accordingly have smaller AHP currents (Wolfart et al., 2001), but expression is more ubiquitous during embryonic development (Sarpal et al., 2004).

SK channel protein subfamily members in humans are encoded by the genes KCNN1, KCNN2 and KCNN3 (Faber and Sah, 2007; Kshatri et al., 2018). Genetic mutations in KCNN3 are associated with risk of disease (Chandy et al., 1998; Gargus et al., 1998; Stöber et al., 1998) but their impact in channel conductance and neuronal function are poorly understood. Mutations in the KCNN3 gene are associated with risk of schizophrenia and bipolar disorder type 1 (Chandy et al., 1998; Gargus et al., 1998; Stöber et al., 1998), as well as of Huntington’s disease and anorexia nervosa (Dallérac et al., 2015; Koronyo-Hamaoui et al., 2002; Saleem et al., 2000).

The KCNN3 gene contains two regions with CAG trinucleotide repeats encoding polyglutamine segments in the protein (Chandy et al., 1998). Because trinucleotide expansions have known pathological impact, the triplet expansion found in the sequence of the KCNN3 gene is of interest. Somewhat surprisingly, an association was found between reduced number of CAG repeats in KCNN3 with schizophrenia (Chandy et al., 1998; Gargus et al., 1998; Ivković et al., 2006; Stöber et al., 1998) but several replication studies failed to confirm the finding (Glatt et al., 2003; Laurent et al., 2003; Li et al., 1998; Ritsner et al., 2003; Saleem et al., 2000; Tsai et al., 1999; Ujike et al., 2001; Wittekindt et al., 1998). Nevertheless, the number of CAG repeats and the differences in allele size may be related with specific disease features, such as time of onset or cognitive performance (Grube et al., 2011; Mitas, 1997).

Cells transfected with transgenic KCNN3 constructs with increasing number of CAG showed a significant reduction in overall conductance and stronger inward rectification (Grube et al., 2011). A 4-base pair deletion in this gene was identified in a patient with schizophrenia and this mutation generated a truncated protein (hSK3Δ) without channel function (Bowen et al., 2001; Miller et al., 2001). In DN, expression of hSK3Δ also suppressed the SK endogenous current and caused increased burst firing activity and increased intracellular calcium signaling (Soden et al., 2013). In this article we provide new data on the impact of known human genetic variations in KCNN3 on its probably channel properties and associated conductance, and discuss possible impact of SK3 channels modifications in DN function.

## Materials and methods

### Data acquisition and sequence alignments

#### Sequence retrieval and alignment

**Figure 1:**
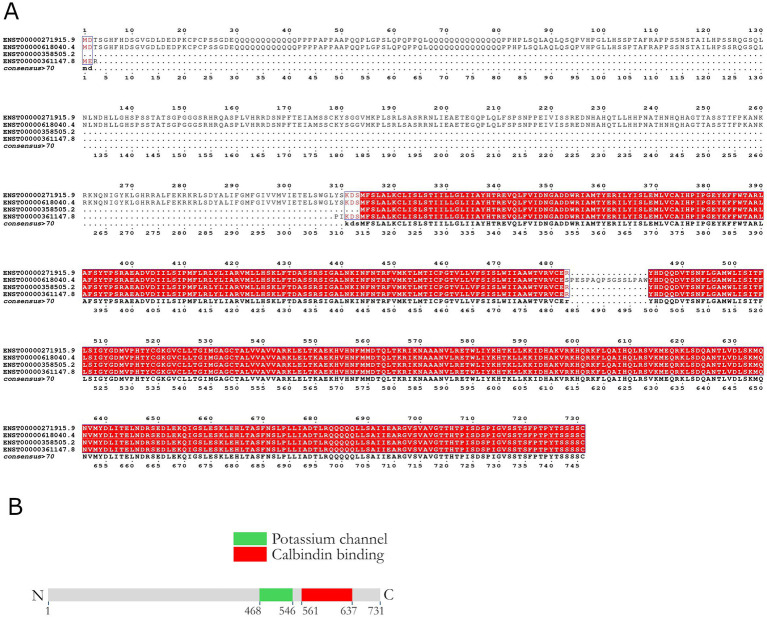
Comparative analysis of KCNN3 splicing variants. **(A)** Sequence alignment of four KCNN3 transcripts reveal two distinct length categories. Long variants (ENST00000271915.9 and ENST00000618040.4) differ only by a 16-amino acid insertion at position 484, while short variants (ENST00000358505.2 and ENST00000361147.8) exhibit a 5-amino acids difference in the region spanning positions 309–314. **(B)** Schematic representation of the coding regions for the main functional domains of the SK3 channel protein.

Alternative splicing variants of the potassium calcium-activated channel subfamily N member 3 (KCNN3) gene were retrieved from the Ensembl database (release 110) (Martin et al., 2023). Multiple sequence alignment of the variant transcripts was performed using T-Coffee version 11.00.8cbe486 with default parameters. The resulting alignments were manually inspected and curated to ensure accuracy, with particular attention to splice junction regions and protein-coding segments (Figure 1).

#### Functional domain analysis

**Figure 2:**
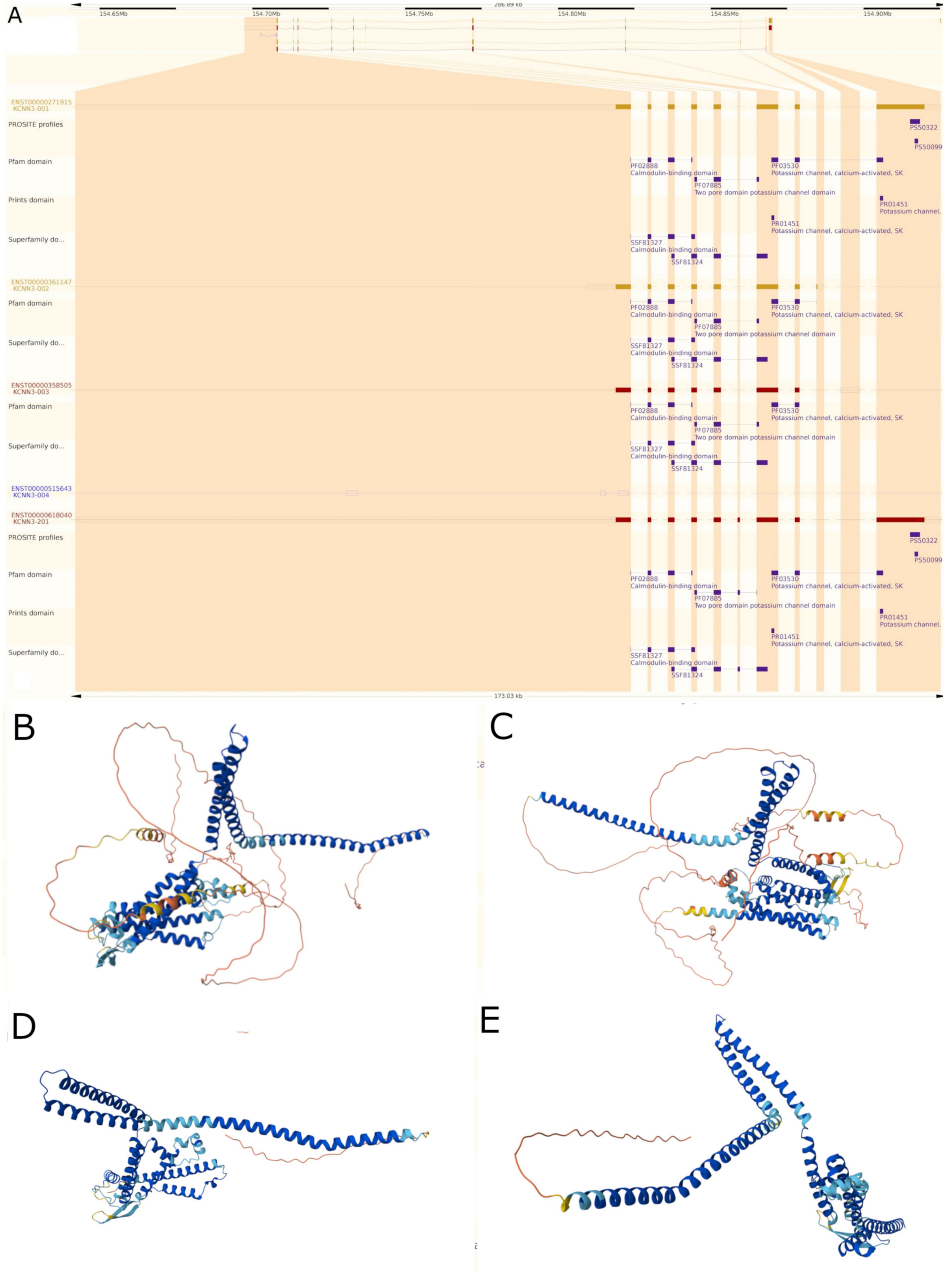
Structural characterization of KCNN3 protein isoforms. **(A)** Domain architecture of KCNN3 (743 amino acids) and its splicing variants. Functional domains and conserved motifs are mapped to their respective positions, with domain boundaries indicated by amino acid position. High-confidence domains (Pfam/SMART identified) are shown in darker colors, while lower-confidence predictions appear in lighter shades. This representation was generated using the Ensembl Protein Feature View tool, integrating data from multiple prediction algorithms and experimental validation. Only domain predictions with significant scores (E-value < 0.001) are included. Annotated protein structure of the human KCNN3 gene product (SK3 channel), highlighting key functional domains across its isoforms. Conserved regions include six transmembrane domains (S1–S6), a pore-forming loop between S5 and S6, and a C-terminal calmodulin-binding domain. Protein domains were mapped using Pfam, PRINTS, PROSITE, and SUPERFAMILY databases. Functional annotations include: PF07885: Two pore domain potassium channel domain; PF03530: Potassium channel, calcium-activated, SK subfamily; PF02888: Calmodulin-binding domain; PR01451/PS00322/PS00909: Signatures of calcium-activated potassium channels. These structural features are critical for the calcium sensitivity and electrophysiological function of SK3 channels. Variability across transcript isoforms may influence channel gating, localization, and regulatory interactions. **(B–E)** Three-dimensional protein structures of KCNN3 splicing variants predicted using the AlphaFold 3 (Abramson et al., 2024). **(B)** Functional variant ENST00000618040.4, **(C)** functional variant ENST00000271915.9, **(D,E)** shorter non-functional variants ENST00000361147.8 and ENST00000358505.2, respectively, revealing the absence of key transmembrane helices in the truncated isoforms (Martin et al., 2023). Renderings were generated in the Human Protein Atlas (https://www.proteinatlas.org/ENSG00000143603-KCNN3/structure+interaction).

Protein domain identification was performed using three complementary databases: Pfam 35.0, SMART 9.0, and InterPro 98.0. Results were cross-referenced to identify conserved and variant-specific domains. Domain architectures were visualized using the Ensembl genome browser protein schematic representation tool (Figure 2). Differences in domain content, organization, and integrity between splicing variants were systematically cataloged.

### Structural model and analysis

#### Reference structure acquisition

**Figure 3:**
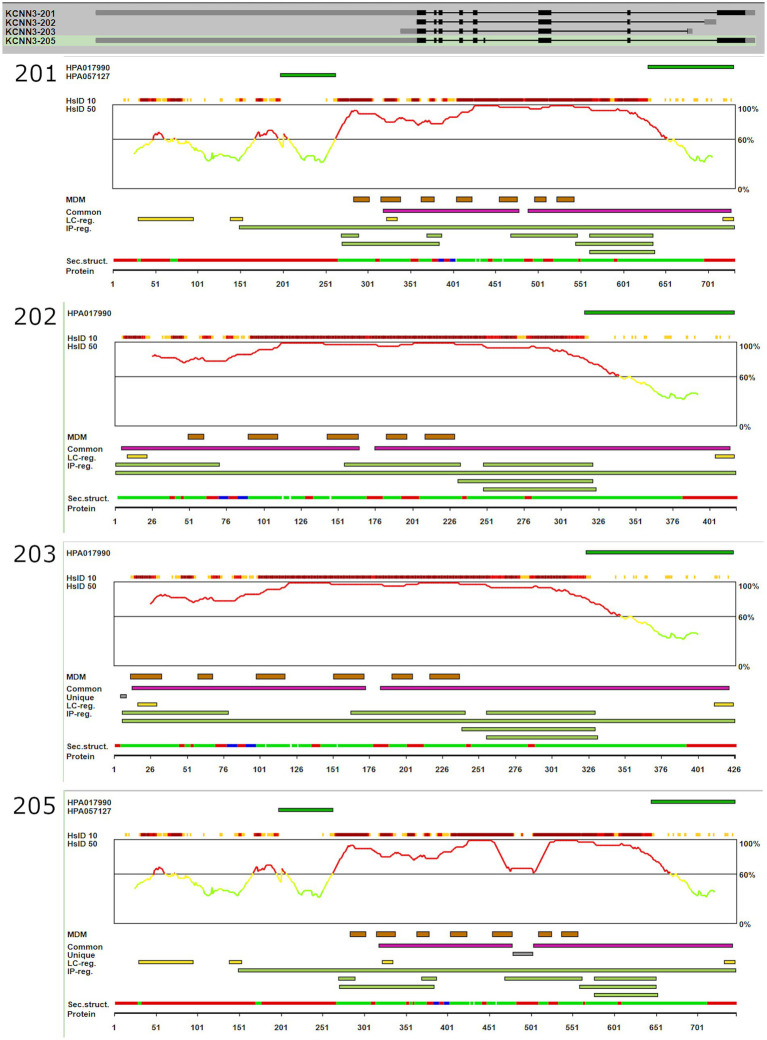
Structural and functional annotation of KCNN3 transcript isoforms. Schematic representation of four transcript isoforms of the KCNN3 gene (KCNN3-201, -202, -203, and -205), including exon-intron structures and corresponding protein features. Top panel: Genomic organization and alternative splicing patterns of the four KCNN3 transcripts. Black boxes represent coding exons; gray lines represent introns. Panels 201–205: Isoform-specific annotations including Antibody target regions (green bars) for antibodies HPA017990 and HPA057127. Sequence alignment reliability from Human Protein Atlas (HsID 10 and HsID 50), where the red-yellow line indicates alignment confidence across the amino acid sequence; Predicted functional and structural features, including: MDM domains (brown); Low complexity regions and intrinsically disordered regions (green and gray); Common and isoform-specific immunogenic regions (purple and orange). Predicted secondary structure elements (alpha helices in red, beta strands in blue, coils in green). Together, these annotations highlight key structural and functional differences among KCNN3 isoforms, which may influence protein stability, localization, and interaction profiles. Data adapted from the Human Protein Atlas (https://www.proteinatlas.org/ENSG00000143603-KCNN3/structure+interaction).

The high-confidence AlphaFold2 (Jumper et al., 2021) model for human SK3 full-length protein (AF-Q9UGI6-F1) was obtained from the AlphaFold Protein Structure Database (Varadi et al., 2024). The canonical KCNN3 sequence (UniProt: Q9UGI6) (Ahmad et al., 2025) served as the reference for comparative analysis. Two-dimensional (Figures 2A, 3) and three-dimensional (Figures 2B–E) structural models of SK3 splicing variants (Variant 202, Variant 203, Variant 205) were predicted using ColabFold (Mirdita et al., 2022), a high-efficiency AlphaFold2 (Jumper et al., 2021) implementation. Structural predictions were performed in batch mode using the following parameters: --model-type alphafold2_ptm, --num-models 5, --num-recycles 3, --rank-by confidence, --use-templates (Abramson et al., 2024), with the wild-type AlphaFold2 model supplied as a template. The top-ranked model based on predicted local distance difference test (pLDDT) scores was selected for each variant.

#### Structure processing and domain annotation

PDB models were parsed using Biopython’s PDBParser (Cock et al., 2009) to verify AlphaFold confidence scores in the B-factor field. Residues with confidence scores below 70 were flagged for cautious interpretation. Functional domains were defined based on literature-curated regions including S1–S6 transmembrane helices, the pore (H5) loop, and the C-terminal calmodulin-binding domain (Supplementary Table 1) (Ahmad et al., 2025).

#### Structural comparison analysis

Cα-based root-mean-square deviation (RMSD) values were calculated using Biopython’s Superimposer for both global (residues 1-731) and domain-specific alignments. Each variant was superimposed onto the AlphaFold2 reference using common residues within each domain. Domain RMSD calculations required ≥5 aligned Cα pairs; otherwise, values were assigned as not available. Per-residue confidence scores (pLDDT) were extracted and mapped to assess regional reliability, with mean pLDDT values reported per domain. Domain integrity was assessed based on completeness of all expected residue indices within each functional region.

### Genetic variant analysis

#### Variant data collection

Single nucleotide polymorphisms (SNPs) and copy number variants (CNVs) across all coding KCNN3 gene splicing variants were analyzed using the Ensembl Variant Effect Predictor (VEP) tool (release 110) (Martin et al., 2023). Variant data were retrieved from gnomAD v3.1.2 (Chen et al., 2024) and ClinVar (Landrum et al., 2014) databases. VEP analysis was performed with parameters: “--species homo_sapiens --assembly GRCh38 --cache --offline --symbol --af --af_1kg --af_gnomad --pubmed --domains --regulatory --biotype --check_existing.” All KCNN3 transcript identifiers (ENST00000618040.4, ENST00000271915.9, ENST00000361147.8, ENST00000358505.2) were specified for targeted analysis.

#### Pathogenicity assessment

Variants were classified according to ACMG/AMP guidelines (Martin et al., 2023; Richards et al., 2015) and filtered to isolate pathogenic and likely pathogenic variants. Non-synonymous amino acid substitutions were prioritized and categorized into four primary types: (1) Alanine to Threonine, (2) Serine to Cysteine, (3) Glycine to Aspartic acid, and (4) Lysine to Glutamic acid conversions. Variant positions were analyzed relative to functional domains identified through domain analysis.

#### Transcript-specific impact analysis

Differential variant impacts across splicing variants were assessed by mapping variant positions to individual transcript protein sequences. Analysis focused on variants affecting conserved functional domains versus transcript-specific regions, with particular attention to differences between long and short SK3 isoforms. Functional consequences were predicted based on amino acid changes, structural location, and cross-species conservation patterns.

## Results

### Overview of variant structures and domains

We analyzed splicing variants and known structural mutations of the gene to predict their functional impact. Currently, there are five known splicing variants of the KCNN3 gene, only four of which are in coding regions. Variants ENST00000618040.4 and ENST00000271915.9 encompass 746 and 731 amino acids, respectively. The other two coding variants, ENST00000361147.8 and ENST00000358505.2 respectively, are much shorter encompassing 426 and 418 amino acids (Figure 1). Variants ENST00000618040.4 and ENST00000271915.9 have a nearly identical sequence, the only difference being a segment insertion in position 484. Variants ENST00000361147.8 and ENST00000358505.2 are also nearly identical, except for positions 309–314. Three dimensional structures obtained using AlphaFold reveal visible differences in the protein conformation as the result of each variant (Figures 2B–E).

### Global structural divergence from the AlphaFold reference model

The global C*α* RMSD values revealed a step-wise divergence among the splice isoforms (Table 1). Variant 202 and Variant 203 produced global RMSD values > 48 Å, whereas Variant_205 deviated by 17.6 Å. The wild type variant (Variant 201) aligned almost perfectly with the AlphaFold model (RMSD ~0 Å), validating the reference structure. We believe that the extreme RMSD of Variants 202/203 is driven primarily by sequence truncations rather than wholesale mis-folding; absent segments inflate RMSD because no equivalent C-α positions exist for superposition.

**Table 1:** Per-domain RMSD mapping: structural elements that are retained or deleted.

Variant	S1–S3	S4	S5–S6 + pore (H5)	CaM-binding domain (561–637)	Comment
WT/AlphaFold	RMSD ≤ 0.4 Å	″	″	Present (pLDDT ≈ 91)	Complete channel bundle
205	RMSD ≤ 0.38 Å	″	″	Present (pLDDT ≈ 91)	Near-WT geometry
203	RMSD ≤ 5.5 Å	9.0 Å	Deleted	Deleted	Partial bundle (S1–S4)
202	RMSD ≤ 5.5 Å	Deleted	Deleted	Deleted	S1–S3 only

No RMS or pLDDT values are reported for domains absent from the input sequence.

Analysis of the short variants and their coding regions with more detail using Ensembl data base (Martin et al., 2023) revealed absence of the motif required for potassium channels (PF03530 Potassium channel, calcium-activated SK) (Figures 2A, 3). In contrast, the two longer variants express all the segments for that motif and are both functional despite the segment insertion. This led us to do a more detailed investigation at the domain structure level.

### Domain-level RMSD analysis and structural integrity

When dissected by domain, all transmembrane (TM) regions (S1–S6) and the pore loop (H5) were preserved in variant 201 and AlphaFold model, as expected (RMSD < 3e−14 Å, all domains present). Variant 205 showed very low RMSD values across all TM domains (≤0.38 Å), indicating a structurally intact pore-forming unit. In contrast, Variant 202 and Variant 203 retained the S1–S3 regions with minimal divergence (RMSD ~0.9–5.5 Å) but exhibited markedly higher RMSD values and missing data across S4, S5, H5, and S6 domains. Specifically; Variant_202 showed complete loss of domains from S4 onwards. Variant 203 retained S4 (RMSD = 9.00 Å) but was missing S5–S6 and the pore loop (Table 1).

### Functional consequences of domain loss across SK3 variants

To contextualize the structural observations, we assessed the functional implications of domain-level disruptions observed in each SK3 isoform. Transmembrane (TM) integrity and the calmodulin-binding domain were evaluated for presence and AlphaFold-predicted confidence (Supplementary Table S1). Wild-Type and AlphaFold models presented all gating and pore motifs intact indicating that likely expected to conduct K^+^ and respond to Ca^2+^. In Variant 205, all TM and regulatory domains were also present, with minor structural deviations from AlphaFold but preserved architecture. Thus, this variant likely retains most functional properties of the wild-type channel, including potassium conductance and calcium sensitivity.

Variant 203 presents a deletion of S5, pore (H5), and S6 domains, which abolished the K + permeation pathway; the CaM-BD region loss removes the Ca^2^ sensor suggesting a disrupted gating and compromised ion selectivity, consistent with severe functional impairment. Lastly, Variant_202 exhibited an even greater truncation (S4-S6 absent) which predicts a complete loss-of-function.

Structural variants were studied in Ensembl in an effort to study the documented mutations that have a known pathogenic effect and further analyzed with SIFT, Polyphen, CADD, REVEL y MetaLR (Martin et al., 2023), eventually settling on SIFT. From the 2,394 reported variants (Supplementary Table S2) 13 variants were identified that were predicted as pathogenic with SIFT (Table 2) and were backed by supportive literature. As shown in Table 2, the 13 pathogenic variants result from missense mutations, in which the resulting protein is largely the same but will encode a different amino acid at a given position due to a nucleotide substitution. In all cases, the consequential effects depend on the specific location and role of the amino acid being changed.

**Table 2:** List of pathogenic variants predicted with SIFT.

Transcript	Variant ID	Location	Alleles	Class	Source	Clinical significance	Consequence type	AA	AA coordinate
ENST00000271915.9	rs2101782564	1:154726011	C/T	SNP	dbSNP	Pathogenic	Missense variant	A/T	536
ENST00000271915.9	rs1571259807	1:154772117	T/A	SNP	dbSNP	Pathogenic	Missense variant	S/C	436
ENST00000271915.9	rs1571260285	1:154772374	C/T	SNP	dbSNP	Pathogenic	Missense variant	G/D	350
ENST00000271915.9	rs1571353663	1:154869160	T/C	SNP	dbSNP	Pathogenic	Missense variant	K/E	269
ENST00000358505.2	rs2101782564	1:154726011	C/T	SNP	dbSNP	Pathogenic	Missense variant	A/T	223
ENST00000358505.2	rs1571259807	1:154772117	T/A	SNP	dbSNP	Pathogenic	Missense variant	S/C	123
ENST00000358505.2	rs1571260285	1:154772374	C/T	SNP	dbSNP	Pathogenic	Missense variant	G/D	37
ENST00000361147.8	rs2101782564	1:154726011	C/T	SNP	dbSNP	Pathogenic	Missense variant	A/T	231
ENST00000361147.8	rs1571259807	1:154772117	T/A	SNP	dbSNP	Pathogenic	Missense variant	S/C	131
ENST00000361147.8	rs1571260285	1:154772374	C/T	SNP	dbSNP	Pathogenic	Missense variant	G/D	45
ENST00000618040.4	rs2101782564	1:154726011	C/T	SNP	dbSNP	Pathogenic	Missense variant	A/T	551
ENST00000618040.4	rs1571259807	1:154772117	T/A	SNP	dbSNP	Pathogenic	Missense variant	S/C	436
ENST00000618040.4	rs1571260285	1:154772374	C/T	SNP	dbSNP	Pathogenic	Missense variant	G/D	350
ENST00000618040.4	rs1571353663	1:154869160	T/C	SNP	dbSNP	Pathogenic	Missense variant	K/E	269

Ensembl was used to study documented mutations known as pathogenic (Martin et al., 2023). All the variants present missense mutations and have been reported in literature. All are deleterious variants with low confidence.

## Discussion

KCNN3 genomic variants have been associated with brain disease and SK channels are expressed widely in brain and play a key role in the control of neuronal activity; thus, drugs modulating SK3 activity may be useful to treat neuropsychiatric disorders (Blank et al., 2004).

SK3 conductance is specifically blocked by apamin and activated by 1-ethyl-1,3-dihydro-2H-benzimidazol-2-one (1-EBIO) and NS309. Another activator, cyclohexyl-[2-(3,5-dimethyl-pyrazol-1-yl)-6-methyl-pyrimidin-4-yl]-amine (CyPPA) is an SK2- and SK3-specific positive modulator (Hougaard et al., 2007). Generally, positive SK3 modulators decrease neuronal activity whereas negative modulation increases firing rate and bursting in neurons (Herrik et al., 2010). Blocking SK3 channels with apamin changes DN pacemaking into a burst firing pattern (Gu et al., 1991; Waroux et al., 2005).

**Figure 4:**
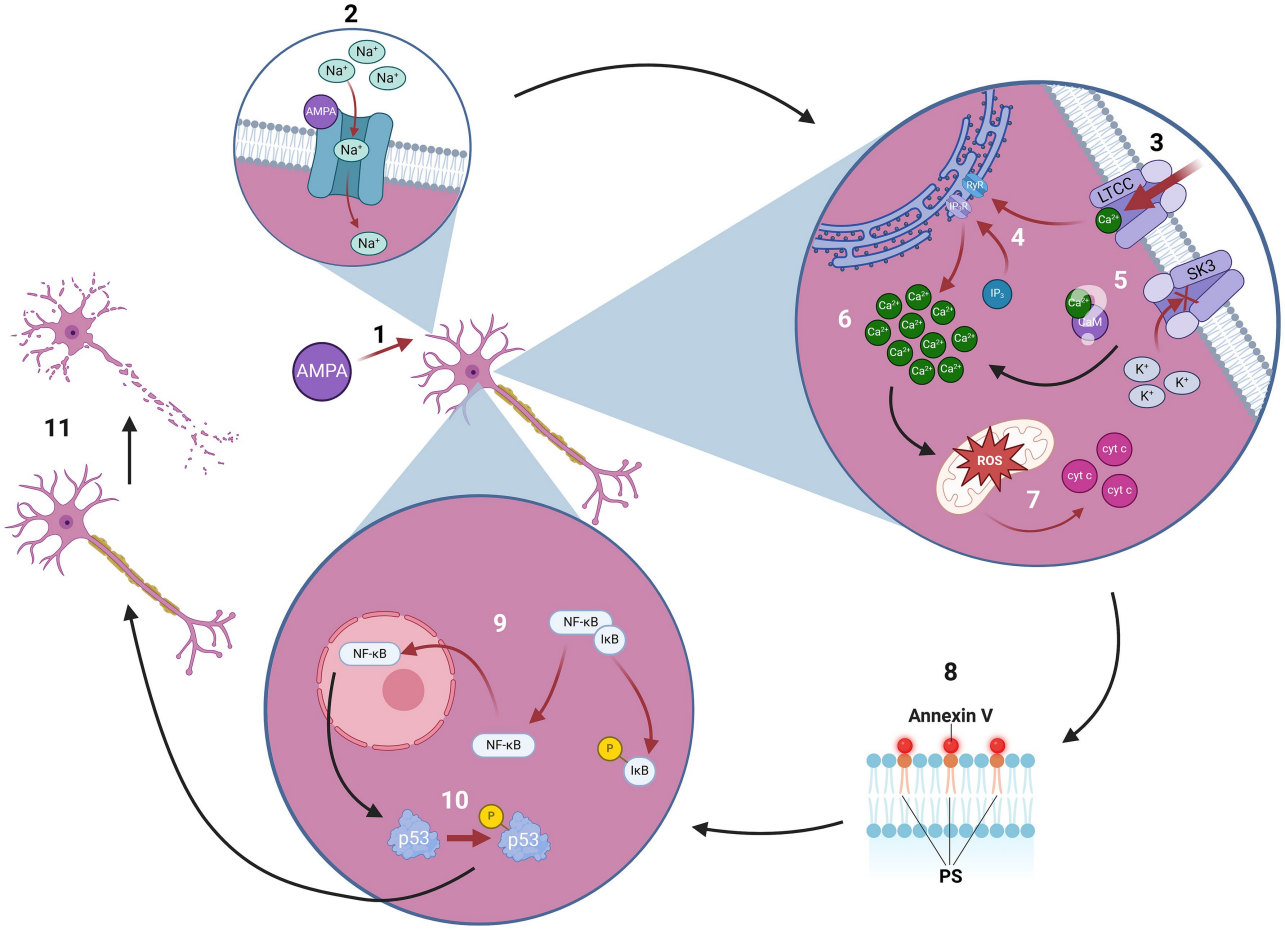
Proposed role of SK3 channels in excitotoxicity-mediated dopaminergic neuronal death: A toxic amount of *α*-amino-3-hydroxy-5-methyl-4-isoxazolepropionic acid (AMPA) contacts the GluR_AMPA_ receptor in dopaminergic neurons (DNs), (2) allowing sodium (Na^+^) into the DNs, causing depolarization in the membrane. (3) This depolarization activates voltage-dependent L-type calcium channels (LTCC), triggering an influx of calcium (Ca^2+^) into the DNs, (4) which further releases intracellular Ca^2+^ stores through the IP_3_ pathway and ryanodine receptors (RyR). (5) At this point in a normal cell an afterhyperpolarization current (I_AHP_) would occur, with potassium (K^+^) exiting the cell through SK3. However, experimental data shows that under AMPA excitotoxicity, the I_AHP_ is significantly reduced or does not take place at all. Meaning that, while the mechanism of this inhibition is not fully understood, it is safe to assume that calmodulin, the calcium-binding protein that activates SK3, is unable to bind normally during AMPA excitotoxicity, which is hypothesized in the above figure. Also, due to their unique sensitivity to AMPA-induced excitotoxicity, DNs are unable to regulate the intracellular levels of Ca^2+^. This creates a (6) destabilization of neuronal ionized Ca^2+^ ([Ca^2+^]_i_) homeostasis, (7) leading to oxidative stress, mitochondrial swelling, increased levels of reactive oxygen species (ROS) and the release of cytochrome C (cyt c). (8) The commitment to die is determined by the phosphatidylserine (PS) translocation to the cellular membrane, which is determined by its attachment to annexin V. (9) After phosphorylation, inhibitor of nuclear factor *κ* B (IκB) is separated from nuclear factor κ B (NFκB) and then degraded. NFκB then translocates to the nucleus, (10) where it activates transcription of protein p53, which later phosphorylates into phos-p53. (11) This is the last step in the dopaminergic neuron-specific programmed death pathway.

In cell cultures of DN, the DN-specific death pathway produced by excitotoxicity (Figure 4), is prevented by SK3 activation with 1-EBIO or CyPPA and facilitated by the SK3 antagonist apamin (Benítez et al., 2011). Likewise, SK channel activation by NS309 confers neuroprotection against oxidative stress with rotenone (Dolga et al., 2014), and 1-EBIO protects DN in organotypic cultures from the neurotoxic effect of 6-hydroxydopamine (6-OHDA) (Wang et al., 2015). In intact animals, 6-OHDA reduces expression of SK3 channels in the substantia nigra pars compacta of the lesioned animals (Mourre et al., 2017).

Mutations in KCNN3 have been associated with schizophrenia (Chandy et al., 1998; Gargus et al., 1998; Stöber et al., 1998). KCNN3 contains two regions with CAG repeats (Chandy et al., 1998) with expansions reported in association with schizophrenia (Mitas, 1997), and with earlier onset of the disease (Ritsner et al., 2003). Other studies found no relation between the CAG trinucleotide repeat and schizophrenia disease or even the onset age (Glatt et al., 2003; Laurent et al., 2003; Li et al., 1998; Saleem et al., 2000; Tsai et al., 1999; Ujike et al., 2001; Wittekindt et al., 1998). A truncated variant of the SK3 with 283 amino acids instead of usual 731 (Bowen et al., 2001) results in a loss of function SK3 channel enhancing the excitability of DN *in vivo* (Soden et al., 2013).

Regarding the gene structure of KCNN3, splicing variants can result in lack of the potassium channel or the calmodulin-binding domain. A truncated SK3 variant similar to a schizophrenia-related mutation did not produce functional channels but selectively suppressed endogenous SK3 currents in a dominant-negative fashion (Tomita et al., 2003). Although the splicing variants ENST00000361147.8 and ENST00000358505.2 are not identical to this truncated variant, the results presented here strongly suggest a similar or identical effect.

The current data consolidate a dual structural/functional role for the Calmodulin-binding domain. Its deletion in Variants 202/203 coincides with wholesale loss of the distal TM bundle (S4-S6) and the pore helix, producing the highest global RMSD (> 48 Å) and predicting non-conductive, Ca^2+^-insensitive channels. Conversely, its retention in WT and Variant 205 correlates with an intact pore and minimal RMSD (<18 Å). These observations extend previous biochemical findings that Calmodulin binding both senses intracellular Ca^2+^ and stabilizes channel architecture. While structurally coherent, the conclusions remain predictive. Post-translational modifications, membrane lipid composition, and protein dynamics are not captured by static in-silico models. Rigorous functional assays are therefore essential before any assertion of clinical impact, particularly regarding neurodegeneration or psychiatric risk, can be made.

We studied in detail 13 documented variants reported in Zimmermann-Laband Syndrome (Bauer et al., 2019; Nam et al., 2023; Schwarz et al., 2022). In further review of the literature, no documented studies were found that focused solely on the possible link of the structural variants of KCNN3 with brain diseases. It is important to note, however, that mutations in KCNN3 that have been related with Zimmermann-Laband are a gain-of-function mutation or mutations that involve a loss of function of the channel have been linked with neurodevelopmental disorders (Nam et al., 2023). Figure 4 provides a model for the role of SK3 channels in dopaminergic neuronal death which may be used as a framework to understand the pathogenicity of channel variants (Maldonado et al., 2020; Benítez et al., 2011; de Erausquin et al., 2003; Dorsey et al., 2006). Further investigation is needed to elucidate the possible effects of the splicing variants in disease is likely to yield new therapeutic targets of possible relevance for Parkinson’s disease, schizophrenia, or both.

## Data Availability

Publicly available datasets were analyzed in this study. This data can be found at: https://www.google.com/url?sa=t&source=web&rct=j&opi=89978449&url=https://www.ensembl.org/index.html&ved=2ahUKEwi7iK6HqrCNAxUWLLkGHWNnGywQFnoECAkQAQ&usg=AOvVaw3m00q2JEfEz-KntGt1NVcQ.
